# Supplementary material for: A set of multi-entry identification keys to African frugivorous flies (Diptera, Tephritidae)
Source: Zookeys. 2014 Jul 24;(428):97–108. doi: 10.3897/zookeys.428.7366 (PMC4143993; doi:10.3897/zookeys.428.7366)
Supplement: Supplementary material 6 — Key to Ceratitis [file zookeys-428-097-s006.zip › SF6_ZooKeys_key to Ceratitis/key/SF6_ZooKeys_key to Ceratitis/Media/Html/Ceratitis silvestrii .htm]

Ceratitis (Ceratalaspis) silvestrii Bezzi


***Ceratitis*** **(*Ceratalaspis*)
*silvestrii* Bezzi**

*Ceratitis silvestrii* Bezzi, 1912: 10.

Body
length: 3.88 (3.75-4.00) mm; wing length: 3.96 (3.75-4.15) mm.

Male

Head.
Antenna yellow. Third antennal segment twice as long as second segment. Arista
with short hairs over entire length. Frons with short scattered hairs which are
the same colour as frons; convex, in lateral view not distinctly projecting
forwards at antennal implant. Face white. Chaetotaxy normal for subgenus,
except dark reddish in colour.

Thorax.
Postpronotum white, unspotted. Ground colour of mesonotum almost white.
Chaetotaxy normal for subgenus, dark reddish. Scapular setae pale. One
anepisternal bristle. Scutellum white basally, otherwise yellow with three
black separate markings apically; basally without spots. Subscutellum
completely dark.

Legs
yellow; setation typical for subgenus, mainly pale especially on femora.
Posterior and posterodorsal rows on fore femur pale. Ventral bristles reddish.

Wing
bands with yellow markings. Banding, marginal band continuous; cubital band
free, medial band absent; crossvein r-m well before middle of discal cell, at
basal third. Crossvein dm-cu posteriorly approximately at same position as
anteriorly.

Abdomen.
Yellow-grey. Setation and banding typical for subgenus.

Female

As
in male except for the following� characters: Mesonotum less white.

(Description
after De Meyer, 1998)
